# Antisocial Learning: Using Learning Window Width to Model Callous-Unemotional Traits?

**DOI:** 10.5334/cpsy.68

**Published:** 2021-05-31

**Authors:** Caroline Moul, Oliver J. Robinson, Evan J. Livesey

**Affiliations:** 1 School of Psychology, University of Sydney, Sydney, Australia; 2 Neuroscience and Mental Health Group, Institute of Cognitive Neuroscience and Research Department of Clinical, Educational and Health Psychology, University College London, London, UK

**Keywords:** psychopathy, callous-unemotional, outcome expectancies, associative learning, learning window width

## Abstract

Psychopathic traits and the childhood analogue, callous-unemotional traits, have been severely neglected by the research field in terms of mechanistic, falsifiable accounts. This is surprising given that some of the core symptoms of the disorder point towards problems with basic components of associative learning. In this manuscript we describe a new mechanistic account that is concordant with current cognitive theories of psychopathic traits and is also able to replicate previous empirical data. The mechanism we describe is one of individual differences in an index we have called, “learning window width”. Here we show how variation in this index would result in different outcome expectations which, in turn, would lead to differences in behaviour. The proposed mechanism is intuitive and simple with easily calculated behavioural implications. Our hope is that this model will stimulate discussion and the use of mechanistic and computational accounts to improve our understanding in this area of research.

With the release of *The Diagnostic and Statistical Manual of Mental Disorders* (5th ed.; *DSM-5*; American Psychiatric Association, 2013), “limited prosocial emotions” was introduced as a specifier to the diagnosis of conduct disorder. “Limited prosocial emotions” describe an individual with high levels of callous-unemotional, or psychopathic, traits. These traits are extremely important in conduct disorder, both clinically and forensically, as they demarcate a significant risk for antisocial behaviour that is serious, resistant to typical treatments, and ultimately an adult diagnosis of antisocial personality disorder. Despite some excellent theoretical advances in recent years, testable mechanistic accounts of psychopathic traits are lacking.

Psychopathic traits are associated with, amongst others, increased risk-taking behaviour, punishment insensitivity, and a diminished conditioned-threat response (Moul, Killcross, & Dadds, 2012). These deficits all point towards an underlying problem with accurately predicting outcomes. Here we propose a testable computational framework, which can explain prior data regarding the role of outcome expectancies in psychopathic traits. We have defined a heuristic risk factor, “Learning Window Width” (W) which refers to the number of trials, or learning episodes, that is used to calculate an average expected outcome for a given cue.

## Defining Learning Window Width

**Figure 1:**
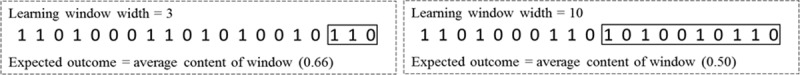
The expected outcomes of an event with base rate occurrence of 0.50 that are generated by learning windows of different widths.

In devising how predictions are affected by a simple learning window, we applied a relatively straightforward formula. Every time a learning episode occurs, for a given context or cue, the outcome is added to the string of previous outcomes that have occurred in the same context or in response to the same cue. Thus, with every learning episode, the list of outcome events experienced grows. The content of that list is then used to form a prediction of the next outcome. However, to account for the influence of the history of recent outcome events, the expected outcome value is calculated from the content of a subset of those events (a window) that includes only the more recent of these past experienced outcomes. This *learning window* begins at the most recently experienced outcome and extends backwards through the history of experiences. The expected value of the outcome on trial t (*EV_t_*) is calculated as a simple mean of the contents of the window (i.e. the sum of all the outcome values divided by the number of observations in the window) as illustrated in ***Figure 1***.

The width of the learning window varies between individuals. As such, one individual may have a narrow window (e.g. averaging the outcomes from the past 3 events to calculate *EV_t_*) whereas another individual may have a wide learning window (e.g. averaging the outcomes from the past 10 events to calculate *EV_t_*). Equation 1 shows how the expected outcome is generated by a learning window width function.


1 \[E{V_t} = \frac{1}{W}\left({\sum\limits_{t - W}^{t - 1} O } \right)\]

Here, *W* represents the learning window width (the number of trials included in the window). The expected value is equal to the sum of the experienced outcomes that fall within the window divided by the learning window width.

In this function *W* has a dual role. When *W* is small, the outcomes from fewer past trials are aggregated for the purposes of estimating the expected outcome by virtue of the role of *W* in the summation term. Concurrently, when *W* is small the relative influence of recent outcomes on the expected outcome is large. In other words, when *W* is small, fewer recent events are used to estimate the mean outcome and thus each of these events has a greater influence on the expected value than if *W* was large.

## Learning Window Width and Reinforcement Learning

W is a simple functional estimate of the breadth of experience that an individual draws upon when making a prediction about a probabilistic event. In this manner, the wider the learning window the less influence each outcome has on the new expected value. In comparison to a narrower learning window, the expectancy generated by a wider window is more robust against stochastic fluctuations in outcomes given a stable base rate. Conversely, a wider learning window is less sensitive to genuine changes in the base rate and a greater number of experiences of the outcome will be required for the expected outcome to approximate the new base rate. The opposite pattern is true for a narrow learning window – expectancies will be influenced *more* by stochastic fluctuations but a stable change in base rate will be adapted to more quickly. W is an explicit instantiation of properties that are implicit in the operations of reinforcement learning models, which use learning rate and decay rate to moderate how quickly predictions change. Behrens, Woolrich, Walton, and Rushworth (2007), for instance, point out that a slow learning rate will result in a long decision history while a fast learning rate will result in a short decision history. They modelled differences in the rate of evidence accumulation by modifying the learning rate in a simple prediction error model (see Supplementary Material A for background information regarding prediction error models and the decay rule).

**Figure 2:**
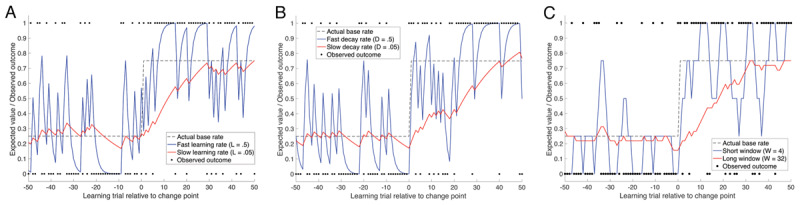
Simulations using prediction error, decay rate, and learning window width as a means of estimating expected value of the outcome (see Supplementary Material A for further information). Example of how a prediction error model (panel **A**), a simple decay rule (panel **B**), and the learning window algorithm (W) (panel **C**) adjusts as a function of stochastic presentations of outcomes and how it adjusts to a sudden change in the base-rate of the outcome. The observed outcome (black circles) is discrete and binary with a value equal to 0 if absent and 1 if present on each trial. At the change point (0 on the x axis), the probability of the outcome shifts from 0.25 to 0.75, as indicated by the black dotted line. The blue and red lines indicate trial-by-trial expected values of the outcome when the rate of learning (panel A), or rate of decay (panel B) is fast (L/D = .5) and learning window width (panel C) is narrow (W = 4) versus when the rate of learning is slow (L/D = .05) or window width is wide (W = 32). Note that expected values on the y axis have been normalized by (1–D)/D for the purposes of illustration in panel B.

Simulations displayed in ***Figure 2*** show that W provides similar predictions to both prediction error and decay rate models in estimating the expected value of an outcome given stochastic variation and changes in the base rate. This correspondence is expected given that W is simply modelling the rate of information loss. It is, however, indifferent to the manner of that loss – whether it is from the overriding effects of new information (prediction error) or from the loss of old information (decay rate), or both. We present the learning window width construct here as a simple computational device for understanding how variations in a fundamental cognitive factor - the breadth of the accumulated evidence that is used to make decisions - might explain clinically relevant variations in behaviour and psychopathology. Our intention at this stage is not to argue that it fits data better than other computational approaches (indeed, it makes similar predictions to delta and decay models), but rather that it provides a complementary, and in many ways simpler, way of conceptualising this cognitive factor. The utility of this approach is that it makes it clear that a single factor (breadth of decision-relevant learning history) can account for a surprising proportion of the variation in behaviour among individuals who differ in terms of their psychopathic traits. Variance in this factor is naturally a corollary of varying parameters related to learning and decay in other models and thus there are a class of models that could make broadly similar predictions at this simple level of analysis. Future work should aim to design tasks to specifically test the predictive value of this simple ‘learning window’ model relative to other more complex models. Indeed one advantage of the current ‘modelling’ approach is that we are explicit about our assumptions and they can be tested and falsified in future work.

Using W we can make clear predictions about behaviour. We propose that behaviours associated with psychopathic (callous-unemotional) traits can be modelled by a wide W. A wide W results in; 1) slower adaptation to a change in the base rate of a cue-outcome contingency, and 2) an enhanced ability to estimate a stable base rate of a cue-outcome contingency and to make predictions that adhere to it despite stochastic fluctuations.

**Figure 3:**
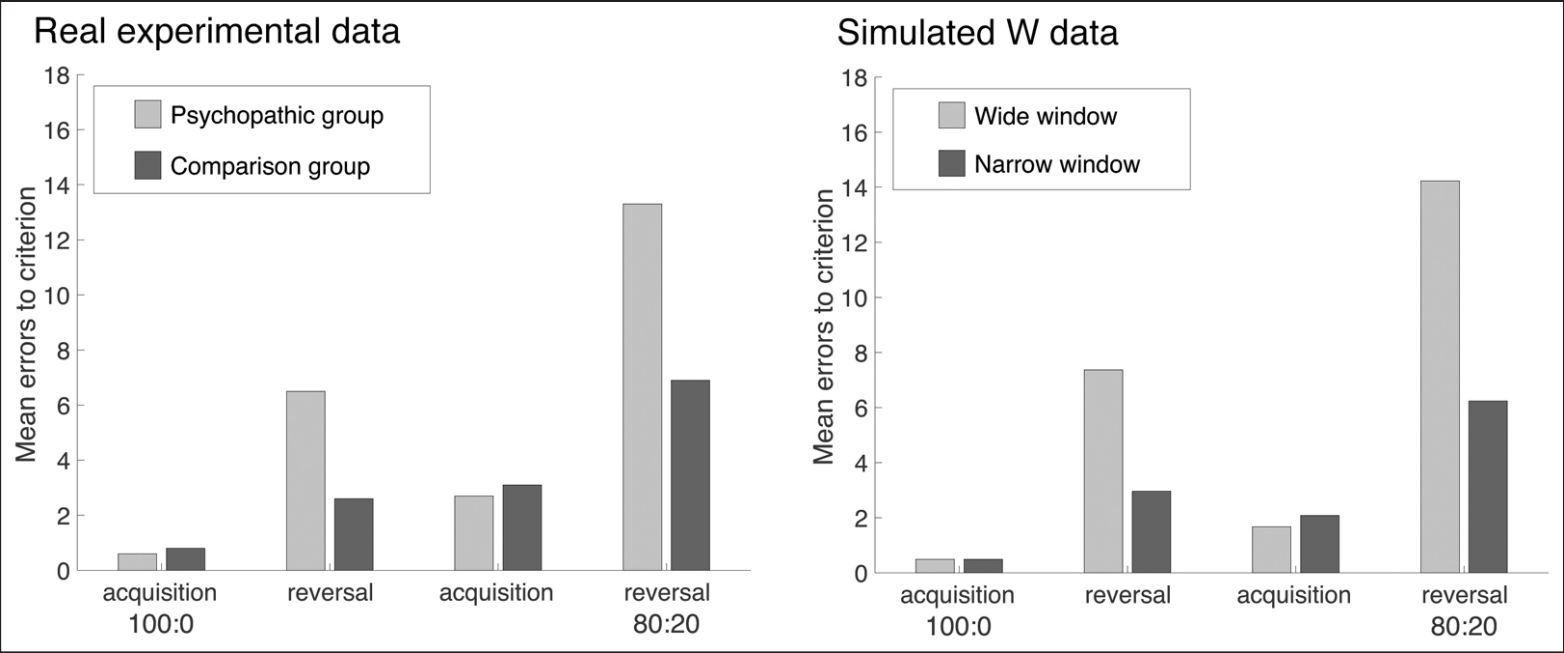
A simulation of the choice task reported by Budhani, Richell, and Blair (2006) using a simple learning window model. For each of two window widths (W = 9, W = 3) we ran 1000 simulated participants on the full 270 trial procedure, assuming an independent learning window for each stimulus, updated whenever the stimulus was chosen and outcome consequently presented (O = 1 when points were won; O = –1 when points were lost). (See supplementary materials B for further details of this simulation) Following Budhani et al. (2006; ***Figure 2***) here we show the mean number of errors to reach a learning criterion of 8 consecutive correct choices, in the initial acquisition and reversal phases, for a 100:0 discrimination and 80:20 discrimination. Simulated data are shown on the right, original empirical data from Budhani et al. (2006) are recreated on the left.

Slower adaptation to a genuine change in a cue-outcome relationship would result in apparent punishment insensitivity. A greater number of punishing trials would be required for a previously rewarded cue to generate a negative expected outcome. This is a simple result of the greater number of trials used to generate the average expected value in a wider window than in a narrower window. Experimentally, people with psychopathic traits demonstrate this deficit in passive-avoidance and response-reversal tasks (Moul et al., 2012). Indeed ***Figure 3*** shows close correspondence between a simulation of the response reversal deficit produced by a wide W and real experimental results from a forensic sample. Importantly for theories that argue for a cognitive, as opposed to an emotional, account of psychopathy, these simulated data were generated by differences in W alone; rewarding and punishing outcomes were treated equally by the simulation.

The model also predicts that a wide W will lead to cue-outcome contingencies appearing predictable even when they include stochastic fluctuations. Risk aversion is the preference to engage in a behaviour that results in a predictable outcome over a behaviour that may lead to a more favourable outcome but for which the likelihood of that outcome is uncertain. The fewer cue-outcome contingencies that are experienced as unpredictable the less uncertainty, and therefore less risk, is perceived. Thus, a wide W would be associated with reduced risk perception and increased behaviour that appears to the observer as risk-taking. Research has found both diminished risk-perception (Hosker, Molnar, & Book, 2016) and increased risk-taking behaviour (Maes, Woyke, & Brazil, 2018) as psychopathic traits increase.

As some researchers have pointed out, the functional window of experiences that people use to make decisions may vary with experience. For instance, Behrens et al. (2007; see also Behrens, Hunt Woolrich, & Rushworth, 2008; Behrens, Hunt, & Rushworth, 2009) hypothesised that people mentally track the *volatility* in an underlying relationship, for example how quickly and predictably the underlying relationship between a cue and an outcome changes over time. They present evidence that when encountering a more volatile set of associations, people will adjust by learning faster, effectively adjusting to using a narrower set of recent experiences to make their decisions about the likelihood of an upcoming event. Similarly, Brazil, Mathys, Popma, Hoppenbrouwers, and Cohn (2017) hypothesised that learners develop mental representations not only of the contingencies between events but also their uncertainty about these representations and hypothesise that differences in representational uncertainty may explain some of the characteristics of psychopathy.

Our approach here is different in that, at least in this first step in developing the model, we are not making any assumptions about the learner’s ability to track volatility or mentally represent uncertainty about the precision of their knowledge. Instead we make the simplifying assumption that W is relatively stable for an individual, or at least that it tends towards a default width that can be relatively wide or narrow. Our aim is not to argue that people are unresponsive to volatility and representational uncertainty, nor that their learning rate is completely fixed—although the simple analysis we present here does not speak to these effects, we do not deny that they are real and important. However, our hypothesis cuts across this issue; what we are asking is whether there is an underlying propensity for some individuals to rely upon a short decision history (fast updating) or a long decision history (slow updating) by default and whether this can explain certain deficits related to psychopathy. Variation in the flexibility of the learning window width (for instance, if some individuals are relatively inflexible, while others change too quickly) could be an avenue to explore.

Future work should test these predictions by creating a reliable and valid test of individual differences in learning window width. Then the specific relationships between W and the behavioural features and empirical findings of psychopathy can be explored, and model comparison/simulations can identify whether learning rate or decay rate, or a combination of the two, best explain the behaviour. Future research should also explicitly test the learning window width model against other models that would make similar predictions. Importantly, these tests would need to be designed to be sensitive to the differences between models so that true comparisons can be made. For example, is psychopathy related to differences in learning rate in volatile situations or differences in the representation of the uncertainty of associations in volatile environments? W makes no claim as to the source of learning window width variation. In other words, processes such as attention to, or the encoding of, outcomes or other latent processes involved in associative learning may have a role to play. Investigating these possibilities is an exciting direction for future work but beyond the scope of this paper.

Importantly, there is no reason to suggest that W is uniquely relevant to psychopathy. Given the use of forensic samples in the majority of psychopathy research, it is possible that W could also be useful in understanding conduct problems more generally. It could also, for example, characterise anxiety, which has often been argued to be the mirror image of psychopathy (Cleckley, 1941). Indeed, enhanced risk-aversion but not loss aversion, has been demonstrated in people with pathological anxiety (Charpentier, Aylward, Roiser, & Robinson, 2017) – the inverse to the psychopathy findings highlighted above. In addition to uncovering the role of individual differences in the generation of outcome expectancies in psychopathy, W may provide insights into any psychiatric condition in which aberrances in perceived unpredictability, risk-perception, and punishment-sensitivity feature. In all, W provides an intuitive, accessible and testable mechanistic account for some of the more challenging and immutable features of psychopathic traits.

## Additional File

The additional file for this article can be found as follows:

10.5334/cpsy.68.s1 Supplementary File. Supplementary Materials A and B.
