# Supplementary material for: Antisocial Learning: Using Learning Window Width to Model Callous-Unemotional Traits?
Source: Comput Psychiatr. 2021 May 31;5(1):54–9. doi: 10.5334/cpsy.68 (PMC11104334; doi:10.5334/cpsy.68)
Supplement: Supplementary File. — Supplementary Materials A and B. [file cpsy-5-1-68-s1.pdf]

Supplementary Material A

Prediction Error Learning Model

For comparison with the learning window model, we used a generalized form of prediction error learning algorithm as shown in Equation A1, as exemplified in past research (Bush & Mosteller, 1953; McClelland and Rumelhart, 1985; Rescorla and Wagner, 1972; Sutto and Barto, 1981; Widrow and Hoff, 1960; Williams, 1992). Predictions are based on the expected value *EV* of the outcome on a given trial *t*, which is updated for the next trial *t+1* according to equation A1.

$$EV_{t+1} = EV_t + L(O_t - EV_t) \qquad \text{(A1)}$$

Here, L is a learning rate parameter (0 < L < 1) that determines how the expected value will change with each new learning episode. Prediction error in the model $(O_t - EV_t)$ is captured by the discrepancy between O_t, the observed outcome (usually, O_t = 1 if outcome present and O_t= 0 if outcome absent) and EV_t, the outcome prediction based on prior learning. Equation A1 is typical of the operations of other prediction error models within the constraints of a task in which the same predictive cues are presented on each and every trial

The speed of learning, as determined by the learning rate L, will affect how quickly associative strength approaches the long-term average when learning about a new set of environmental predictors is in its infancy or when the probability of the outcome changes. But fast learning does not improve the model's ability to settle around a long-term average. In fact, in certain respects, a *slower* rate of learning may be optimal for tracking a relatively stable base-rate. Provided the learner has experienced sufficient episodes to approximate the probability of the event occurring in the future, it is undesirable to quickly adjust one's predictions when

chance would dictate that an outcome is experienced more frequently or less frequently than the long-term average for a short run of trials.

Accumuluative Learning and Decay Model

The prediction error algorithm described above models changes in associative value that are assumed to be stable in the long term; once learned, they are not forgotten unless new experiences interfere with them. Information acquired about the likelihood of the outcome on trial *t can* be lost, but only by virtue of it being over-written by new information from trials $t+1$, $t+2$ and so forth. An alternative approach, one with clear parallels in terms of its simple predictions, is to assume that learning about outcomes is not limited by prediction error, but instead decays over time as a process of time-dependent “forgetting”, is used in existing literature (Erev, 1998; Yechiam and Busemeyer, 2005; Yechiam and Ert, 2007). In this sense, information acquired about the likelihood of the outcome on trial N is *necessarily* lost at an incremental rate on trials $t+1$, $t+2$, etc. One can model a decay function of this sort using the following equation:

$$EV_{t+1} = (1 - D).EV_t + O_t \quad \text{(A2)}$$

Here, D represents a decay rate such that $0 < D < 1$. In Equation A2, only a proportion of previously learned associations, captured by (1 – D), is retained, overlaid by a new memory of the learning episode on the current trial.

Cumuluative decay algorithms of the sort shown in Equation A2 assume that learning consists of tallying up experienced outcomes, but gradually “forgetting” those outcomes over time. According to this decay rule, fast forgetting leads to fast information loss for reasons that are reasonably intuitive. Therefore it is perhaps not surprising that when the model has a high value for D, predictions are more strongly based on what has just occurred. More generally, and

like the prediction error model, the decay rule displays a recency bias and the strength of this bias depends on the rate of decay.

Supplementary Material B: Discrete Predictions and their Relationship to Past Trials

Many of the types of tasks for which formal models of learning are most useful involve learning about and predicting the occurrence of multiple outcomes, or choosing among multiple possible actions. Learning rules can easily be generalised to such situations but require a further formal step to convert expected values into discrete predictions or choices that vary probabilistically across trials. In this context, discrete choices are often simulated using the softmax function (see Luce, 1959), which converts expected values for a number of behavioural options into choice probabilities.

$$P\left|C_j\right| = \frac{e^{k \cdot EV_j}}{\sum_1^{N(j)} e^{k \cdot EV_j}} \qquad \text{(B1)}$$

The softmax function shown here in equation B1 calculates the probability of choosing each option from a set of *j* choices (the probabilities of all choices summing to 1). The probability of each choice is determined by its expected value and by an inverse temperature parameter *k*. Higher values of *k* lead to more consistent choice of the option with the highest *EV*, whereas with lower values of *k*, the choice probabilities become increasing similar. For the simulation of the Budhani et al., (2006) experiment, the softmax function was used with k = 10.

References – Supplementary Materials A and B

Bush, R. R., & Mosteller, F. A. (1953). Stochastic Model with Applications to Learning. *Annals of Mathematical Statististics*; *24*(4), 559-585.

Erev, I., & Roth A. E. (1998) Predicting How People Play Games: Reinforcement Learning in Experimental Games with Unique, Mixed Strategy Equilibria. *The American Economic Review*, *88*(4), 848-881.

Luce, R.D. (1959) *Individual choice behavior*. Oxford, England: John Wiley.

McClelland, J. L., & Rumelhart, D. E. (1985). Distributed memory and the representation of general and specific information. *Journal of Experimental Psychology: General*, *114*(2), 159-197.

Rescorla, R. A., & Wagner A. A. (1972). Theory of Pavlovian conditioning: The effectiveness of reinforcement and non-reinforcement. *Classical Conditioning: Current Research and Theory*

Sutton, R. S., & Barto, A. G. (1981). Toward a modern theory of adaptive networks: Expectation and prediction. *Psychological Review*, *88*(2), 135-170.

Widrow, B., & Hoff, M. E. (1960). *Adaptive switching circuits*. New York: IRE.

Williams, R. J. (1992). Simple statistical gradient-following algorithms for connectionist reinforcement learning. *Machine Learning*, *8*(3), 229-256.

Yechiam, E., & Busemeyer, J. R. (2005) Comparison of basic assumptions embedded in learning models for experience-based decision making. *Psychonomic Bulletin & Review*, *12*(3), 387-402.

Yechiam, E., & Ert, E. (2007). Evaluating the reliance on past choices in adaptive learning models. *Journal of Mathematical Psychology*, *51*(2), 75-84.
